# Characterization of Electromechanical Delay Based on a Biophysical Multi-Scale Skeletal Muscle Model

**DOI:** 10.3389/fphys.2019.01270

**Published:** 2019-10-09

**Authors:** Laura Schmid, Thomas Klotz, Tobias Siebert, Oliver Röhrle

**Affiliations:** ^1^Chair for Continuum Biomechanics and Mechanobiology, Institute for Modelling and Simulation of Biomechanical Systems, University of Stuttgart, Stuttgart, Germany; ^2^Department of Motion and Exercise Science, Institute of Sport and Motion Science, University of Stuttgart, Stuttgart, Germany; ^3^Stuttgart Center for Simulation Sciences (SC SimTech), University of Stuttgart, Stuttgart, Germany

**Keywords:** injury, neuromuscular control, biomarker, skeletal muscle modeling, tendon, continuum mechanics, *in silico*

## Abstract

Skeletal muscles can be voluntary controlled by the somatic nervous system yielding an active contractile stress response. Thereby, the active muscle stresses are transmitted to the skeleton by a cascade of connective tissue and thus enable motion. In the context of joint perturbations as well as the assessment of the complexity of neural control, the initial phase of the muscle-tendon system's stress response has a particular importance and is analyzed by means of electromechanical delay (EMD). EMD is defined as the time lag between the stimulation of a muscle and a measurable change in force output. While EMD is believed to depend on multiple structures / phenomena, it is hard to separate their contributions experimentally. We employ a physiologically detailed, three-dimensional, multi-scale model of an idealized muscle-tendon system to analyze the influence of (i) muscle and tendon length, (ii) the material behavior of skeletal muscle and tendon tissue, (iii) the chemo-electro-mechanical behavior of the muscle fibers and (iv) neural control on EMD. Comparisons with experimental data show that simulated EMD values are within the physiological range, i.e., between 6.1 and 68.6 ms, and that the model is able to reproduce the characteristic EMD-stretch curve, yielding the minimum EMD at optimal length. Simulating consecutive recruitment of motor units increases EMD by more than 20 ms, indicating that during voluntary contractions neural control is the dominant factor determining EMD. In contrast, the muscle fiber action potential conduction velocity is found to influence EMD even of a 27 cm long muscle by not more than 3.7 ms. We further demonstrate that in conditions where only little pre-stretch is applied to a muscle-tendon system, the mechanical behavior of both muscle and tendon tissue considerably impacts EMD. Predicting EMD for different muscle and tendon lengths indicates that the anatomy of a specific muscle-tendon system is optimized for its function, i.e., shorter tendon lengths are beneficial to minimize the neural control effort for muscles primary acting as motor in concentric contractions.

## 1. Introduction

Skeletal muscles can provide active resistance to joint perturbations by developing protective tension. However, the active response of the muscle is delayed by sensory organs, the neural system, and the muscle's rate of force development. The delay intrinsically caused by the muscle-tendon system is typically quantified by means of electromechanical delay (EMD). In detail, EMD is defined as the time lag between the (initial) stimulation of a muscle at the neuromuscular junction and a measurable change in force output (Cavanagh and Komi, 1979). EMD is believed to depend on joint angle, motor unit recruitment, the mechanical tissue properties (of both muscle and tendon), the muscle fiber action potential (AP) conduction velocity, and the excitation-contraction coupling pathway (cf. e.g., Cavanagh and Komi, 1979; Hopkins et al., 2007; Nordez et al., 2009). Further, gender, age, physical activity, fatigue, muscle temperature, and pathological conditions showed to influence EMD (cf. e.g., Yavuz et al., 2010; Cè et al., 2013; Esposito et al., 2016; Sözen et al., 2019). EMD is associated with injuries; for example, an increased EMD is associated with the enhanced risk of anterior cruciate ligament (ACL) injury in women (cf. e.g., Moore et al., 2002; Blackburn et al., 2009) as well as ankle sprain (Hopkins et al., 2009). Further, representing a delay in the control pathway, EMD can be used as a biomarker, determining the complexity of neuromuscular control tasks. Consequently, a better understanding of the factors influencing EMD contributes to the development of prevention, treatment, and rehabilitation strategies for ankle instability (Hopkins et al., 2009).

In humans, experimental studies utilizing different joints and various examination methods observed EMD ranging from 8 ms to more than 100 ms (Blackburn et al., 2009; Grosset et al., 2009; Ateş et al., 2019). However, since experimental studies are limited by the applicable boundary conditions and the observable physical quantities, it is challenging to quantify which property / structure contributes how much to EMD (Cavanagh and Komi, 1979; Nordez et al., 2009). In this context numerical experiments offer the possibility to study knockout conditions and systematically vary the model parameters. By employing biophysical models, one can attribute each state variable to specific structures / components and thus, simulations can predict the influence of individual factors and contribute to the development and improvement of treatment strategies for pathological conditions that are affected by EMD.

To the best knowledge of the authors, the work of Mörl et al. (2012), which employs Hill-type muscle models, is the only simulation study investigating EMD in skeletal muscles. However, Hill-type muscle models are spatially lumped and thereby oversimplifying many structural and functional properties. Whenever heterogeneities in structural or functional properties, e.g., propagation of APs through muscle tissue, are essential to investigate EMD, Hill-type muscle models are not capable of doing so. Thus, the aim of this work is to apply a biophysically detailed, three-dimensional, chemo-electro-mechanical model to simulate EMD and thereby identify the major factors influencing EMD. Therefore, the continuum-mechanical multi-scale model of Heidlauf, Röhrle and co-workers is used (Röhrle et al., 2008, 2012; Heidlauf and Röhrle, 2013, 2014; Heidlauf et al., 2016, 2017) and adapted with respect to the constitutive material description of passive skeletal muscle tissue and tendinous tissue.

## 2. Methods

The multi-scale skeletal muscle model represents the basis of the proposed work. Thus, section 2.1 provides a brief summary on the general modeling framework. In order to reproduce the mechanical material behavior of both passive skeletal muscle tissue and tendon tissue, we modified the continuum-mechanical constitutive description of the passive muscle tissue and tendon tissue with respect to previous works (cf. section 2.1.1). In section 2.2 we describe the *in silico* experiments that are used to analyze the influence of (i) muscle and tendon length, (ii) the macroscopic material properties, (iii) the excitation-contraction coupling pathway and (iv) neural control on EMD.

### 2.1. The Multi-Scale Skeletal Muscle Model

In summary, the chemo-electro-mechanical multi-scale skeletal muscle model (Heidlauf and Röhrle, 2013, 2014; Heidlauf et al., 2016, 2017) solves for the macroscopic deformations in response to neural stimulation and the applied boundary conditions. Thereby, the model resolves the functional organization of skeletal muscle tissue in motor units (a motor unit consists of a motor neuron and all muscle fibers it innervates) as well as the propagation of action potentials along muscle fibers and excitation-contraction coupling. A three-dimensional continuum model is applied to simulate the mechanical behavior on the macroscopic tissue scale. Therefore, the (quasi-static) balance of linear momentum, i.e.,

(1) $$\begin{array}{l} \mathrm{div}P\;=\;0\;,\;\text{in }\Omega_{\text{M}}, \end{array}$$

is solved. Therein, ***P*** is the first Piola-Kirchhoff stress tensor and Ω_M_ is an arbitrary continuum body, representing within this work both skeletal muscle and tendon tissue. In order to solve Equation 1 the mechanical material behavior, i.e., the macroscopic stress tensor ***P***, needs to be constitutively defined. It is assumed that both skeletal muscle tissue and tendinous tissue are incompressible. Tendinous tissue is modeled as hyper-elastic material. By the linear superposition of a passive, hyper-elastic contribution ***P***_passive_ and the activation modulated (sarcomere based) contributions ***P***_active_ and ***P***_titin_, i.e.,

(2) $$\begin{array}{l} P\;=\;P_{\text{passive}}+P_{\text{active}}+P_{\text{titin}}-pF^{-T}\;, \end{array}$$

one obtains the common constitutive material description for skeletal muscle tissue. The hydrostatic pressure *p* enters the equation as a Lagrange multiplier to enforce the incompressibility condition (cf. e.g., Holzapfel, 2000), **F** is the deformation gradient tensor and (*)^−*T*^ is the transposed inverse of (*). While the passive stress tensor, ***P***_passive_, is based on a phenomenological constitutive model (cf. section 2.1.1), a biophysical multi-scale modeling approach describes the muscle's active behavior (cf. section 2.1.2).

#### 2.1.1. Constitutive Modeling of Passive Muscle Tissue and Tendon Tissue

**Figure 1:**
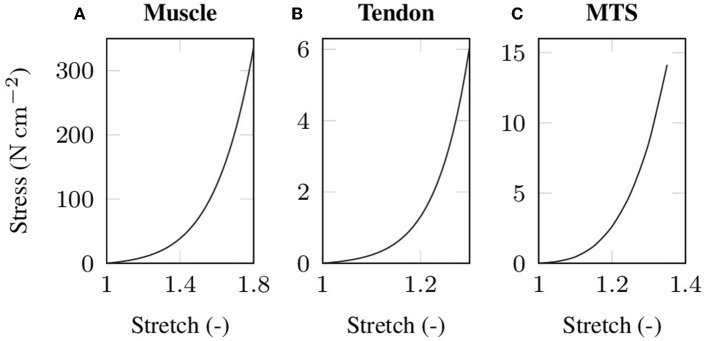
Stress-stretch data under uniaxial tension as used in the baseline experiment for **(A)** passive skeletal muscle tissue and **(B)** tendon tissue. Note that the tendon tissue is more compliant than the muscle tissue to compensate for the unphysiologically high cross-sectional area of the tendon that arises from the idealized cuboid geometry. The simulated stress-strain behavior of the baseline muscle-tendon system (MTS) under uniaxial tension is shown in **(C)**.

While the focus of the previous works of Heidlauf, Röhrle and co-workers (Heidlauf and Röhrle, 2013, 2014; Heidlauf et al., 2016, 2017) was to simulate skeletal muscle tissue, EMD is associated with the interaction between muscle and tendon tissue, i.e., tendon stretch induced by muscle contraction. In order to simulate EMD the mechanical behavior of tendinous tissue needs to be adequately resolved by the continuum-mechanical constitutive model. Thereby, the characteristic J-like stress-strain relation, which can be observed for both passive skeletal muscle tissue and tendon tissue during uniaxial extension, can be well described by an isotropic, hyper-elastic Ogden-type material (Ogden, 1972). The corresponding strain-energy density function reads:

(3) $$\begin{array}{l} W_{\text{Ogden}}\;=\;\mu_{0}\overset{N}{\underset{i=1}{\sum }}\frac{\mu_{i}}{\alpha_{i}}\left(\lambda_{1}^{\alpha_{i}}+\lambda_{2}^{\alpha_{i}}+\lambda_{3}^{\alpha_{i}}-3\right), \end{array}$$

where λ_1_, λ_2_, λ_3_ denote the principal stretches and μ_0_, μ_*i*_ and α_*i*_ (*i* = 1, 2, …, *N*) are material parameters. Assuming *N* = 1, the hyper-elastic tendon stress / the passive contribution of skeletal muscle tissue's stress response (cf. Equation 2) yields:

(4) $$\begin{array}{l} P_{\text{passive}}=2F\frac{\partial W_{\text{Ogden}}}{\partial C}\\ \;=2\mu F\overset{3}{\underset{k=1}{\sum }}\frac{1}{\lambda_{k}}\lambda_{k}^{\alpha -1}\left(m_{k}⊗m_{k}\right)\;, \end{array}$$

where **m**_*k*_ denote the eigenvectors corresponding to the eigenvalues $e_{k}=\lambda_{k}^{2}$ (*k* = 1, 2, 3) of the right Cauchy-Green deformation tensor **C**. The material parameters μ_m_, α_m_, μ_t_ and α_t_ for both passive muscle and tendon tissue, respectively, are chosen such that the stiffness ratio between a muscle and a tendon of equal cross-sectional area corresponds to the experimentally determined stiffness ratio of Calvo et al. (2010). Table 1 lists the material parameters and Figure 1 illustrates the resulting (uniaxial) stress-stretch relations. Note that due to the microscopic fiber structure, skeletal muscle tissue is commonly assumed to be transversely isotropic. However, there is no clear scientific evidence which is the preferred direction of the tissue. While some experiments showed enhanced stiffness in the muscle fiber direction (cf. e.g., Morrow et al., 2010), others reported the cross-fiber direction to be stiffer (cf. e.g., Böl et al., 2012; Takaza et al., 2013). Thus, assuming isotropic material behavior seems to be as good as any other choice of transversal isotropy.

**Table 1:** Model parameters: values for the baseline experiment and their modifications.

**Description**	**Symbol**	**Value (baseline / modifications)**	**References**
Resting length of the muscle-tendon system	$l_{\text{mts}}^{0}$	4.5/27/54 cm	Siebert et al., 2014a Arnold et al., 2010
Muscle-tendon length ratio	MTR	0.33/0.5*/0.66*	Siebert et al., 2017
1^st^ muscle material parameter	μ_m_	2.224/2.798*/1.285* N cm-2	Calvo et al., 2010
2^nd^ muscle material parameter	α_m_	8.536/8.641*/9.182* (–)	Calvo et al., 2010
1^st^ tendon material parameter	μ_t_	0.041/0.097*/0.016* N cm-2	Calvo et al., 2010
2^nd^ tendon material parameter	α_t_	19.06/18.47*/19.78* (–)	Calvo et al., 2010
Maximum active stress	*p*_max_	10 N cm-2	Eddinger et al., 1986
Maximum contraction velocity	*v*_max_	0.005 cm ms-1	Eddinger et al., 1986
Attached post-power stroke state during isometric tetanic contraction	$A_{\text{max}}^{2}$	0.044456 (–)	
Membrane capacitance	*C*_m_	1/1.98*/0.58 μF cm-2	Shorten et al., 2007
Surface-to-volume ratio	*A*_m_	500 cm-1	Röhrle et al., 2012
Conductivity	σ	3.828 mS cm-1	Röhrle et al., 2012
Average distortion caused by a power-stroke	*x*_0_	8 ×10-3 μm	Barclay, 1998
Reciprocal speed of recruitment of motor units (MU)	–	0*/3.5*/6.5/10* ms MU^-1^	Vecchio et al., 2019

Values referenced by “*” are chosen arbitrarily to illustrate general dependencies.

#### 2.1.2. Multi-Scale Modeling of Active Muscle Tissue

To describe the activation modulated properties of muscle tissue, i.e., ***P***_active_ and ***P***_titin_ (cf. Equation 2), a biophysical multi-scale modeling approach is chosen. Microscopically, skeletal muscles' active mechanical behavior is governed by the complex interplay of the sarcomere proteins actin and myosin (cf. Huxley and Hanson, 1954; Huxley and Niedergerke, 1954; Huxley, 1957) as well as titin (cf. e.g., Lindstedt and Nishikawa, 2017; Herzog, 2018). Thereby, the active force response is modulated by electrical signals. In short, when a muscle fiber is stimulated at the neuromuscular junction an AP propagates along the muscle fiber and triggers calcium release from the sarcoplasmic reticulum, serving as a second messenger enabling cross-bridge cycling and modulating the mechanical properties of titin. In order to relate the biophysical behavior of a muscle fiber to the macroscopic deformations of the tissue, one-dimensional computational muscle fibers are embedded into the three-dimensional continuum body representing muscle tissue. The propagation of APs and excitation-contraction coupling within the muscle fibers is modeled by employing the monodomain model, i.e.,

(5a) $$\begin{array}{l} \frac{\partial V_{\text{m}}}{\partial t}=\frac{1}{A_{\text{m}}C_{\text{m}}}\frac{\partial }{\partial s\left(F\right)}\left(\sigma \frac{\partial V_{\text{m}}}{\partial s\left(F\right)}\right)\\ \;-\frac{1}{C_{\text{m}}}I_{\text{ion}}\left(y,V_{\text{m}},I_{\text{stim}}\right),\;\text{in}\Omega_{\text{F}}, \end{array}$$

(5b) $$\begin{array}{l} \;\dot{y}\;=\;f\left(y,F,\dot{F},V_{\text{m}},t\right),\text{in}\Omega_{\text{F}}\;. \end{array}$$

Therein, *V*_m_ is the transmembrane potential, *C*_m_ denotes the capacitance of the cell membrane, *A*_m_ the membrane surface-to-volume ratio, *I*_ion_ is the overall current density crossing the cell membrane, *I*_stim_ represents an externally applied stimulus, *s* is the spatial variable along the path of a muscle fiber and Ω_F_ is a one-dimensional continuum body representing a muscle fiber. Further, **y** is a vector describing all state variables related to excitation-contraction coupling. The evolution of the cellular state variables can be described by a set of coupled ordinary differential equations, which are summarized in Equation (6b). In detail, the applied cell model incorporates a phenomenological description of the muscle fibers' cell membrane (Aliev and Panfilov, 1996), which is coupled to a simplified Huxley-type model (Razumova et al., 1999; Campbell et al., 2001) simulating the active stresses γ induced by cross-bridge cycling (cf. Heidlauf et al., 2017), i.e.,

(6) $$\begin{array}{l} \gamma \left(l_{\text{hs}},y\right)\;=\;f_{\text{l}}\left(l_{\text{hs}}\right)\frac{x_{1}A_{1}+x_{2}A_{2}}{x_{0}A_{2}^{\text{max}}}\;. \end{array}$$

Therein, *l*_hs_ is the half-sarcomere length, *f*_l_ is the force-length relation, which is the piece-wise linear sarcomere based function obtained by Gordon et al. (1966), *A*_1_ and *A*_2_ are the number of cross-bridges in the attached pre-power stroke state and post-power stroke state, respectively, and *x*_1_ and *x*_2_ are the corresponding cross-bridge displacements. Further, $A_{2}^{\text{max}}$ is the number of cross-bridges in the attached post-power stroke state during a tetanic contraction and *x*_0_ is the displacement of post-power stroke cross-bridges during an isometric contraction. To simulate the microscopic titin induced stresses θ, we follow Heidlauf et al. (2016, 2017) and solve the biophysical sticky-spring model proposed by Rode et al. (2009). Thereby note that the titin stresses are history dependent and are modulated by the intracellular calcium concentration (for details see Rode et al., 2009; Heidlauf et al., 2016, 2017).

Considering the multi-scale skeletal muscle model, all sub-models show different characteristic length and time scales. In order to limit the computational costs different discretization methods (both in space and time) are utilized for the different sub-models (for a detailed description of the numerical model see Heidlauf and Röhrle, 2013). For example, while the numerical solution of the monodomain model (cf. Equation 5a) requires a relatively fine spatial resolution, which is discretized by employing linear Lagrangeian finite elements, a coarser finite element mesh is used to solve for the continuum-mechanical problem, which is discretized by employing Taylor-Hood elements based on tri-linear and tri-quadratic Lagrangeian shape functions. In order to couple the muscle fiber models and the three-dimensional continuum mechanical problem, microscopic quantities, i.e., representing state variables of the muscle fiber models, need to be homogenized and macroscopic quantities, i.e., the state variables of the three-dimensional continuum model, are interpolated. In detail, to evaluate the macroscopic stress tensor (cf. Equation 2) at the gauss points of the three-dimensional muscle mesh, both the microscopic cross-bridge stresses, $T_{\text{H}}:\gamma \to \bar{\gamma }$, and titin stresses, $T_{\text{H}}:\theta \to \bar{\theta }$, need to be homogenized (Heidlauf and Röhrle, 2013), where (*) denote microscopic quantities, $\left(\bar{*}\right)$ denote homogenized macroscopic quantities and *T*_H_ denotes the corresponding homogenization operator. Further, the muscle deformations, i.e., reflecting the half-sarcomere lengths, are interpolated by the shape functions of the three-dimensional finite element discretization and evaluated at the node points of the muscle fiber meshes. Finally, the macroscopic cross-bridge and titin-induced stress tensors can be summarized to read:

(7a) $$\begin{array}{l} P_{\text{active}}=p_{\text{max}}\bar{\gamma }F\left({\text{a}}_{0}⊗{\text{a}}_{0}\right) \end{array}$$

(7b) $$\begin{array}{l} P_{\text{titin}}\;=\;p_{\text{max}}\bar{\theta }F\left({\text{a}}_{0}⊗{\text{a}}_{0}\right)\;. \end{array}$$

Therein, **a**_0_ is a unit vector in the muscle fiber direction in the reference configuration and *p*_max_ is a material parameter representing the maximum isometric tension.

### 2.2. *In silico* Experiments

All simulations are performed by utilizing the open-source software library OpenCMISS (Bradley et al., 2011). The implementation of the multi-scale model is adopted from previous works and thus, details on the general computational framework can be found e.g., in Heidlauf and Röhrle (2013) and Heidlauf et al. (2017). Note that only the calculation of the stress tensor needs to be adapted in order to represent the constitutive model described in section 2.1.1.

**Figure 2:**
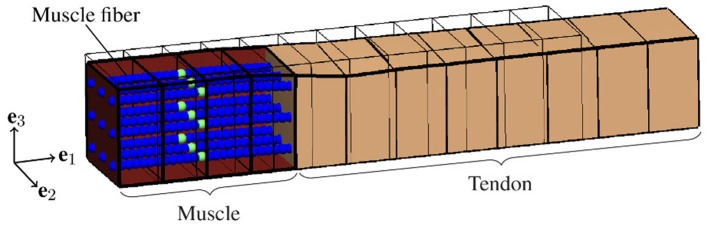
Illustration of the idealized muscle-tendon system utilized for the baseline experiment at the time of stimulation, i.e., *t* = 0ms, and an external stretch of $\bar{\lambda }=1.25$. A set of representative computational muscle fibers (illustrated by spheres) is embedded into the muscle tissue (red). Thereby, the virtual neuromuscular junctions, i.e., the points of stimulation, are marked in green. The tendon is represented by the beige cuboid on the right side. Further, the thin black lines represent the reference configuration, i.e., $\bar{\lambda }=1$ and no activation of the muscle fibers.

**Figure 3:**
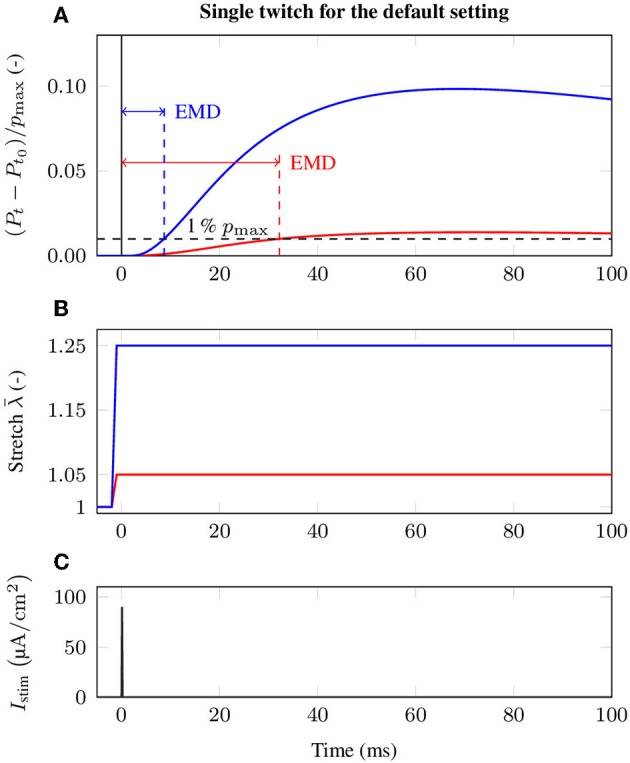
Exemplary stress response during the baseline experiment induced by a single twitch stimulation for two different initial stretches, i.e., $\bar{\lambda }=1.05$ (red) and $\bar{\lambda }=1.25$ (blue). In detail, the figure shows **(A)** the change of the normalized stress (*P*_*t*_ − *P*_*t*_0__)/*p*_max_ and evaluation of EMD, **(B)** the applied stretch $\bar{\lambda }$ in the *e*_1_-direction and **(C)** the external stimulation current density *I*_stim_ at the neuromuscular junction, illustrating the event of activation.

There is strong evidence that EMD depends on the applied stretch (cf. e.g., Muraoka et al., 2004; Nordez et al., 2009). In order to analyze the relation between EMD and the externally applied stretch $\bar{\lambda }:=l_{\text{mts}}/l_{\text{mts}}^{0}$ for a specific muscle-tendon system, a set of isometric single twitch experiments is performed. Starting from the undeformed reference configuration with length $l_{\text{mts}}^{0}$, the muscle-tendon system is passively stretched to a desired length *l*_mts_, yielding seven equally distributed initial stretches $\bar{\lambda }$ between 1.05 and 1.35. This stretch range corresponds e.g., to the range of motion of the rat tibialis anterior muscle (Hawkins and Bey, 1997). After stretching the muscle-tendon system to the desired length, a contraction is induced by prescribing the stimulation patterns to the mid-point of each muscle fiber (cf. Figure 3), i.e., by applying a stimulation current density *I*_stim_ of 90 μA cm-2 (cf. Figure 2). The resulting reaction forces at the right end of the tendon (cf. Figure 2) are recorded and used to calculate the nominal stresses, i.e., the reaction forces with respect to the cross-sectional area in the reference configuration. Finally, EMD is calculated as the time difference between the initial stimulus of (an arbitrary subset of) the muscle fibers and the instant when the recorded stress increased by 1 % of the maximum active stress *p*_max_ (Mörl et al., 2012) (cf. Figure 3A).

#### 2.2.1. The Baseline Experiment

Based on variations of model parameters, which are associated with specific structures and phenomena, we use the biophysical multi-scale model to predict the contribution of individual structures and phenomena to EMD. To ensure comparability of all *in silico* experiments, each representing the muscle-tendon system in a specific condition based on a specific set of parameters (cf. Table 1), we employ a baseline experiment (cf. Figure 2) serving as a benchmark. The baseline experiment considers an idealized muscle-tendon system, i.e., a muscle fiber bundle which is attached to a tendon, both with a cuboid shape and a constant cross-sectional area of 1 cm2 (cf. Figure 2). Thereby, influences of the geometry on the simulation results, e.g., caused by complex muscle architectures, can be excluded. Stimulating all muscle fibers synchronously evokes the contraction. Starting from the described baseline experiment scenario, we introduce the following model modifications.

#### 2.2.2. Influence of Muscle and Tendon Length on EMD

To capture the relative length of muscle and tendon, the measure of muscle-tendon length ratio (MTR) is introduced, i.e., MTR$\;:=\;l_{\text{m}}^{0}/l_{\text{mts}}^{0}$. EMD is evaluated for an isolated skeletal muscle, i.e., no tendon is attached to the muscle, and compared to muscle-tendon systems with different overall lengths $l_{\text{mts}}^{0}$ as well as different MTRs. Thereby, all major length ratios, i.e., muscle longer than tendon, muscle shorter than tendon and equal length of muscle and tendon, are captured.

#### 2.2.3. Influence of the Mechanical Properties of Tendon and Passive Muscle Tissue on EMD

Beside the length of muscle and tendon, EMD should be characterized with respect to the internal properties of muscle as well as tendon tissue. Thereby, both passive skeletal muscle tissue and tendinous tissue show J-like stress-strain behavior (cf. Figure 1), i.e., a flat toe zone is followed by a nearly linear segment, and thus significant stresses can only be transmitted after stretching the material beyond the flat region of the toe zone. The length of the initial toe zone is believed to be an important property when characterizing EMD. Thus, the toe zone as well as the slope of the stress-stretch relation is modulated for both the passive mechanical skeletal muscle behavior and the mechanical behavior of the tendon.

#### 2.2.4. Influence of the Chemo-Electro-Mechanical Properties of the Muscle Fibers on EMD

Considering the active contractile behavior of skeletal muscle tissue, the influence of the AP conduction velocity, excitation-contraction coupling, stretch modulated calcium sensitivity of force regulation and the muscle fibers' twitch properties on EMD are investigated. Changing the capacitance of the cell membrane *C*_m_ modulates the AP conduction velocity. Note that also other parameters such as the tissue's conductivity σ or the muscle fibers' surface-to-volume ratio *A*_m_ determine the AP conduction velocity. An AP triggers the release of calcium-ions from the sarcoplasmic reticulum serving as a second messenger and controlling cross-bridge cycling. Thereby, it is known that the calcium sensitivity depends on the applied stretch, i.e., given a constant calcium concentration increasing the applied stretch leads to higher relative sarcomere forces (cf. e.g., Rack and Westbury, 1969; Balnave and Allen, 1996). This behavior is incorporated into the cell model by modulating the calcium concentration at half saturation of the troponin binding sites, Ca_50_, i.e.,

(8) $$\begin{array}{l} {\text{Ca}}_{50}\;=\;{\text{Ca}}_{50}^{0}\text{exp}(b\left(1-l_{\text{hs}}\right))\;, \end{array}$$

where ${\text{Ca}}_{50}^{0}$ and *b* are parameters that need to be fitted to reproduce experimental data. Note that *b* = 0 indicates no length dependent calcium sensitivity. By choosing, for example, ${\text{Ca}}_{50}^{0}=1$ and *b* = 8.3986, one can reproduce the behavior reported in Balnave and Allen (1996). Finally, the twitch response of a specific muscle fiber is known to depend on multiple factors such as the muscle fiber type and the properties of the associated motor unit. To simulate the influence of the twitch shape on EMD, the reaction rates of the cross-bridge dynamics model (Razumova et al., 1999) are altered by a multiplier yielding faster / slower twitch responses, which are characterized by the resulting time-to-peak values.

#### 2.2.5. Influence of Neural Control on EMD

Recruiting all muscle fibers at the same time is an appropriate method to investigate EMD caused by the internal properties of the muscle-tendon system. However, during voluntary contraction (*in vivo*) also the behavior of the neural system and the conjunct functional organization of the neuromuscular system into motor units affects the evolution of active tension and thus EMD measurements. In order to resolve skeletal muscles' functional organization, i.e., incorporating motor units into the model, the number of the (computational) muscle fibers is increased to 225 and the fibers are randomly grouped into ten motor units (cf. e.g., Röhrle et al., 2012). The properties of the motor units are chosen based on basic physiological principles, i.e., Henneman's size principle (Henneman et al., 1965). Accordingly, the number of the (computational) muscle fibers per motor unit is distributed exponentially and the motor units are recruited from smallest to largest. Within this work, both the influence of the recruitment strength, i.e., determining how many motor units are active, and the influence of the motor unit synchronicity on EMD measurements are analyzed.

## 3. Results

### 3.1. The Baseline Experiment and Stretch Dependence of EMD

**Figure 4:**
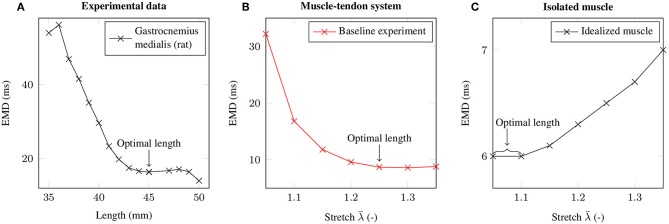
**(A)** Mean EMD values based on isometric experiments of the rat gastrocnemius medialis at different muscle-tendon lengths (Siebert et al., 2014a,b, 2016). **(B)** EMD-stretch curve for the baseline experiment. **(C)** EMD-stretch curve for an isolated muscle (i.e., without tendon). The respective optimal lengths are labeled with an arrow.

By employing the baseline experiment scenario, i.e., a 1.5 cm long muscle that is attached to a 3 cm long tendon, the computationally determined EMD-stretch curve shown in Figure 4B is qualitatively similar to the EMD-stretch curve derived from experimental measurements of Siebert et al. (2014a,b, 2016) (cf. Figure 4A), i.e., a steep decrease for stretches below the optimal length of the muscle-tendon system that plateaus at stretches beyond the optimal length. In contrast, simulating EMD for the isolated muscle, i.e., without a tendon, yields higher EMD values for stretches above the optimal muscle length. Thereby note that the optimal length is shifted toward higher stretches for the muscle-tendon system. For the baseline muscle-tendon system the highest recorded EMD value (32.2 ms) is attained for an applied stretch of $\bar{\lambda }=1.05$. The lowest EMD value (8.6 ms) is obtained for an applied stretch of $\bar{\lambda }=1.3$. In contrast, the EMD-stretch curve for the isolated muscle reaches a minimum of 6 ms at optimal muscle / sarcomere length, which ranges from $\bar{\lambda }=1.05$ to $\bar{\lambda }=1.1$. Further, EMD increases slightly up to 7 ms for higher stretches.

### 3.2. Influence of Muscle and Tendon Length on EMD

**Figure 5:**
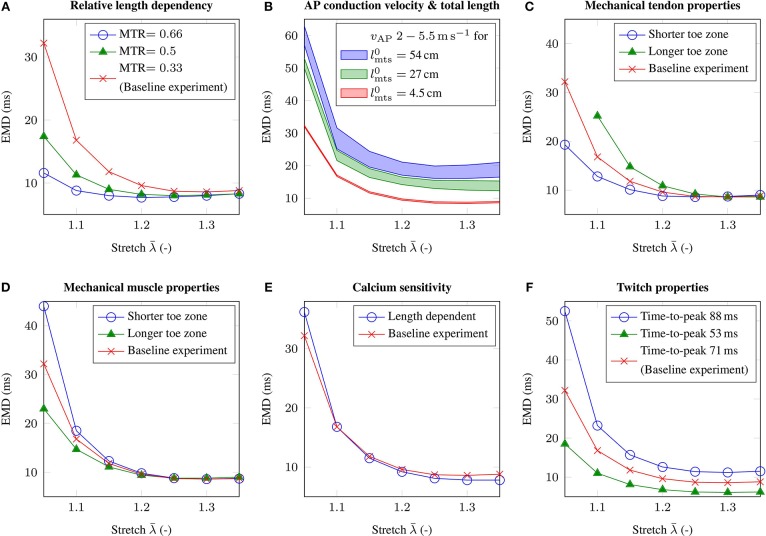
The figure shows the EMD-stretch curves for parameter variations addressing **(A)** the muscle-tendon length ratio MTR, **(B)** the total length of the muscle-tendon system $l_{\text{mts}}^{0}$ and the AP conduction velocity *v*_AP_, **(C)** the material behavior of the tendon, **(D)** the passive material behavior of skeletal muscle tissue, **(E)** length dependent Ca^2+^-sensitivity and **(F)** the twitch properties of the muscle fibers. Thereby, all parameter variations are compared to the baseline experiment (red).

Geometrically, the idealized muscle-tendon system is fully characterized by its overall length and the relative length of the muscle or the tendon. Increasing the relative length of the muscle while fixing the overall length of the muscle-tendon system, i.e., applying different MTRs, yields similar shaped EMD-stretch curves as for the baseline experiment. However, considering low stretches, the obtained EMD values are much smaller for shorter relative tendon lengths (cf. Figure 5A). Further, increasing the overall size of the muscle-tendon system to 54 cm while maintaining the MTR, both increases EMD on the plateau region of the EMD-stretch curve and increases the slope of the initial segment of the EMD-stretch curve (cf. Figure 5B).

### 3.3. Influence of the Mechanical Properties of Tendon and Passive Muscle Tissue on EMD

Beside depending on the lengths of a muscle-tendon system, EMD is also believed to be sensitive to the material properties of passive skeletal muscle tissue and tendinous tissue. Considering the mechanical material behavior of tendinous tissue, shortening the toe zone of the tendon's characteristic stress-stretch relation has a similar effect as reducing the relative size of the tendon, i.e., for low stretches EMD decreases by up to 12.9 ms (cf. Figure 5C). Further, in contrast to modulating the MTR, EMD increases slightly for higher initial stretches. Increasing the length of the toe zone of the tendon's stress-stretch relation yields the opposite behavior, i.e., higher EMD values can be observed for low stretches. Thereby note that considering the muscle-tendon system with a longer toe zone of the tendon's stress-stretch relation, the change in stress does not exceed the EMD threshold of 1 % *p*_max_ for an applied stretch of $\bar{\lambda }\;=\;1.05$.

Similar results can be observed when simulating altered mechanical material properties of passive skeletal muscle tissue. In detail, shortening the toe zone of the muscle's passive stress-stretch relation yields higher EMD values for applied stretches of $\bar{\lambda }\leq 1.25$. Comparing the muscle-tendon system from the baseline experiment and the muscle-tendon systems reflecting altered tissue properties, the maximum difference in EMD is 11.8 ms (cf. Figure 5D).

### 3.4. Influence of the Chemo-Electro-Mechanical Properties of the Muscle Fibers on EMD

The AP conduction velocity determines how fast an external stimulus, which is during physiological conditions determined by the firing times of the motor neurons, innervates the whole muscle fiber. Adjusting the membrane capacitance of the muscle fibers *C*_m_ modulates the AP conduction velocity. While for the baseline experiment the AP conduction velocity is 3.5 m s-1, we employ parameter variations yielding AP conduction velocities of 2.0 and 5.5 m s-1, respectively. Further, since the transport of information is closely related to the spatial dimensions of a specific muscle-tendon system, we consider muscle-tendon systems with three different lengths and the same MTR. Considering the geometry of the baseline muscle-tendon system, i.e., $l_{\text{mts}}^{0}=$ 4.5 cm, it can be shown that the effect of the AP conduction velocity on EMD is negligible, i.e., <0.5 ms (cf. Figure 5B). However, increasing the overall length of the muscle-tendon system, i.e., $l_{\text{mts}}^{0}=$ 27 cm and $l_{\text{mts}}^{0}=$ 54 cm, leads to higher EMD values for all applied external stretches. The difference between EMD for the highest AP conduction velocity and EMD for the lowest AP conduction velocity increases to 3.7 and 6.4 ms, respectively.

Considering excitation-contraction coupling, modeling length modulated calcium sensitivity on the cellular level (cf. Equation 8), increases EMD by 4 ms for an applied stretch of $\bar{\lambda }=1.05$, however reduces EMD on the plateau region of the EMD-stretch curve. Thereby, the observed changes are in the range of 1 ms (cf. Figure 5E).

Finally, the influence of the muscle fibers' twitch properties on EMD is analyzed. While the time-to-peak for the baseline muscle-tendon system's muscle fibers is 71 ms, assuming a muscle consisting purely of rather slow-twitch muscle fibers, i.e., which are characterized by a time-to-peak of 88 ms, increases EMD by up to 20.3 ms. In contrast, simulating a muscle consisting exclusively of muscle fibers with a time-to-peak of 53 ms, yields in a decrease in EMD, which is up to 13.7 ms for an applied stretch of $\bar{\lambda }=1.05$ (cf. Figure 5F).

### 3.5. Influence of Neural Control on EMD

**Figure 6:**
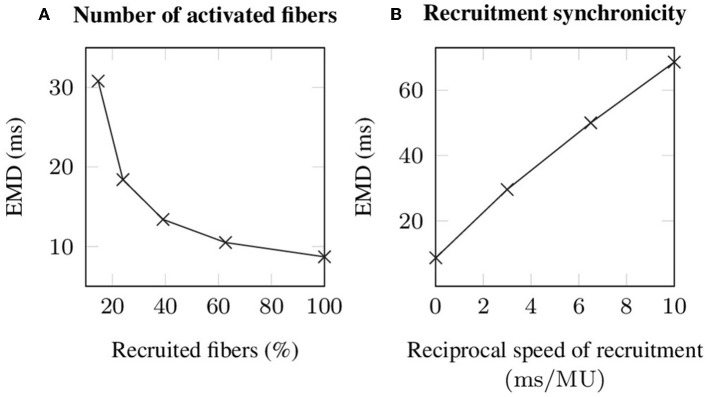
Simulated EMD **(A)** in dependence of the number of recruited muscle fibers, whereby all stimulated fibers are recruited synchronously and **(B)** during progressive recruitment, whereby the motor units (MU) are recruited from the smallest to the largest and with a constant time interval between the recruitment of two consecutive motor units. Note that for all simulations an external stretch of $\bar{\lambda }=1.25$ is applied.

During voluntary contractions, EMD is not purely determined by the properties of the muscle-tendon system, but considerably influenced by neural control (Hopkins et al., 2007). Thereby, particularly the number of recruited motor units, as well as the speed of recruitment, i.e., reflecting the time difference between the recruitment of two motor units and thus neural synchronicity, are important characteristics affecting EMD measurements. Since the observed changes in EMD caused by different recruitment patterns are nearly constant for all applied stretches, i.e., the observed changes are smaller than 3 ms, the corresponding results are only shown for an arbitrarily chosen stretch of $\bar{\lambda }=1.25$, which reflects the optimal length of the baseline muscle-tendon system. Thereby, increasing the number of recruited muscle fibers from 14.67 % (corresponding to the recruitment of 6 out of 10 motor units) to 100 % decreases EMD by 22.1 ms (cf. Figure 6A). Note that recruiting <14.67 % of the fibers is not sufficient to exceed the utilized EMD threshold. Further, simulating progressive recruitment of the motor units based on Henneman's size principle, i.e., from the smallest to the largest motor unit, while prescribing a constant delay of 3, 6.5, and 10 ms between the recruitment of two consecutive motor units, increases EMD for a stretch of 1.25 by 20.9 to 59.9 ms (cf. Figure 6B).

## 4. Discussion

Within this work, EMD was characterized by employing a detailed biophysical multi-scale model of an idealized muscle-tendon system. The multi-scale model is capable to qualitatively reproduce experimentally determined EMD-stretch curves. Thus, it can be concluded that the multi-scale model considers the major structures and phenomena contributing to EMD. By employing parameter variations, the model can contribute to obtain a profound comprehensive understanding of EMD. Note that the characteristic EMD-stretch curve, i.e., a steep decrease for stretches below the optimal length of the muscle-tendon system that plateaus for stretches beyond the optimal length, can only be reproduced when considering the serial arrangement of a muscle and the tendon and thus it can be concluded that EMD has to be considered as a property of a muscle-tendon system and not only of a muscle.

### 4.1. Model Limitations

While all simulated EMD values are in the range between 6.1 and 68.6 ms and thus well reflect experimental findings (Blackburn et al., 2009; Grosset et al., 2009), a quantitative comparison of the simulation results and specific experimentally determined EMD values is beyond the scope of this study. Due to the high diversity of both muscle and tendon tissue a single set of parameters is not representative at all. Further, existing experimental studies are hardly comparable since they differ with respect to the analyzed joint / muscle-tendon system, the applied stimulation method and the position control of the joint / the muscle-tendon system. Thus, within that work we aim to establish generic relationships between EMD and multiple structural and functional properties of both skeletal muscle and tendon tissue. To avoid influences of the geometry on the obtained results an idealized cuboid geometry is chosen. However, due to the geometrical flexibility of the utilized finite element method, the analysis of more complex / realistic anatomical conditions is feasible (cf. e.g., Röhrle et al., 2012; Heidlauf and Röhrle, 2013). Thereby note that simulating anatomically more complex geometries can lead to significant computational costs that require efficient numerical methods as well as the use of a (highly) parallel computing environment (cf. Bradley et al., 2018). Further, due to the lack of knowledge on both the mechanical and the geometrical properties of aponeurosis tissue, i.e., representing the transition between muscle and tendon tissue, an aponeurosis is not considered within the presented simulations. The identification of consistent model parameters is a general issue when applying complex biophysical multi-scale models. However, the observation that the model output is consistent with multiple experimental findings rises confidence that the multi-scale model of the muscle-tendon system realistically reflects the real physiological system. Both viscous material behavior and inertia effects have been neglected as their influence is expected to be small during isometric contractions (cf. e.g., Günther et al., 2012; Ross et al., 2018). Moreover, simulating other phenomena that are believed to influence EMD, such as the temperature, gender, age, training, and pathological conditions (cf. e.g., Yavuz et al., 2010; Cè et al., 2013; Esposito et al., 2016; Sözen et al., 2019), is beyond the capabilities of the utilized model.

### 4.2. *In silco* Characterization of EMD for an Idealized Muscle-Tendon System

#### 4.2.1. Influence of Muscle and Tendon Length on EMD

It is known that the MTR is closely related to the function of a muscle-tendon system, i.e., while muscles with a short relative tendon length often have primary motor functions, muscles with a long relative tendon length typically act as energy storage (Biewener, 1998; Mörl et al., 2016). Further, MTR evolves during aging (Siebert et al., 2017). By varying the MTR, it can be demonstrated that the relative muscle and tendon lengths have a considerable influence on EMD, i.e., a higher MTR reduces EMD (cf. Figure 5A). Therefore, it can be speculated that shorter relative tendon lengths, which are associated with muscle-tendon systems primary acting as a motor during concentric contractions, are beneficial to minimize the neural control effort. Increasing the total length of the muscle-tendon system is leading to higher EMD values for all applied stretches. This behavior implies that the neuromuscular control as well as the development of protective tension during joint perturbations is more challenging in lager species / subjects. Note that EMD in larger scaled muscle-tendon systems can potentially be reduced by an optimization of the muscle architecture, e.g., based on pennated muscle fiber arrangements or by the appearance of in series arranged muscle fibers (Loeb et al., 1987; Heron and Richmond, 1993; Lacourpaille et al., 2013). Further, in muscles constituted of long muscle fibers the dependency of EMD on the AP conduction velocity increases (cf. Figure 5B). However, the increase in EMD due to a higher muscle length is smaller than the additional time the AP requires to reach the boundary of the longer fibers. This implies that the muscle-tendon system generates force before all sarcomeres within a fiber are activated. In conclusion, the contribution of the AP conduction velocity to EMD is complex and can not be estimated by simple geometrical considerations and a given AP conduction velocity as e.g., proposed by Mörl et al. (2012).

#### 4.2.2. Influence of the Mechanical Behavior of Tendon and Passive Muscle Tissue on EMD

The passive material behavior of skeletal muscle tissue and the material behavior of tendinous tissue is highly diverse and can be remodeled due to training or caused by pathological conditions. Assuming a shorter toe zone of the tendon's characteristic stress-stretch relation leads to reduced EMD for stretches bellow $\bar{\lambda }=1.25$ (cf. Figure 5C). The marginal increase in EMD for higher stretches is most likely caused by increased relative muscle fiber stretches, yielding reduced force production capabilities, which, in turn, can be explained by the force-length relation. Experimentally, Kubo et al. (2001) showed that isometric training can enhance the stiffness of the tendon, leading to shorter EMD, which is in agreement with the presented results. Accordingly, this illustrates a possible prevention strategy to reduce EMD in the context of joint perturbations. However, note that within the work of Kubo et al. (2001) EMD was only measured for a single joint angle.

Considering skeletal muscles' passive material behavior, a shorter toe zone of the muscle's passive stretch-stress relation yields higher EMD values for stretches smaller than $\bar{\lambda }=1.25$ (cf. Figure 5D). Thereby note that the assumed altered material parameters lead to a higher slope of the muscle's passive stress-stretch relation for all muscle lengths. Thus, for a given activation, the muscle's steady-state contractile length, i.e., the muscle length where the passive and the active muscle stresses cancel each other out, is shifted toward the reference configuration. Consequently, if compared to the baseline parameters, higher active stresses are required to stretch the tendon to a specific length, yielding increased EMD. While remodeling of the passive mechanical behavior of muscle tissue can be caused by physical training (Grosset et al., 2009), enhanced passive muscle stiffness is also associated with fibrotic muscle tissue, which is often caused by pathological conditions such as e.g., stroke, muscle trauma, muscular dystrophy, or cerebral palsy (cf. e.g., Lieber and Ward, 2013; Yucesoy et al., 2015; Chapman et al., 2016). Thus, besides determining the range of motion, the passive material behavior of skeletal muscle tissue has a notable influence on EMD and hence on muscle and motor control. While the presented results suggest that enhanced passive muscle stiffness increases EMD (cf. Figure 5D), previous studies (Grosset et al., 2009; Longo et al., 2017) reported that an increased muscle stiffness reduces EMD. However, while Grosset et al. (2009) estimated the passive muscle stiffness by performing quick release tests, i.e., the obtained stiffness value is smeared by the contribution of the tendon, Longo et al. (2017) simultaneously decreased both the muscle and tendon stiffness. Thus, both studies failed to investigate the effect of changes in muscle tissue stiffness in an isolated manner. Further, both mentioned studies calculated a single stiffness value, which is not sufficient to characterize a complex non-linear material such as skeletal muscle tissue. In summary, it can be concluded that EMD is closely related to the passive mechanical muscle behavior in response to compression, i.e., determining the steady-state contraction length of a muscle for a given activation. Unfortunately, non-invasive measurements of skeletal muscles' stress-strain behavior are hardly feasible. Therefore, biophysical detailed constitutive modeling frameworks taking into account microscopic properties of the tissue (cf. e.g., Bleiler et al., 2019) could be a valuable methodology to further analyze EMD with respect to various physiological / pathological conditions.

#### 4.2.3. Influence of the Chemo-Electro-Mechanical Properties of the Muscle Fibers on EMD

The twitch properties of muscle fibers are highly diverse and depend on the muscle fiber type, the properties of the associated motor unit as well as the function of the muscle. Further, the twitch dynamics can change due to fatigue, aging or physical training. Within this work, the force response of the muscle cells is simulated by using a biophysical cross-bridge dynamics model (cf. Razumova et al., 1999; Heidlauf et al., 2017). Thereby, the twitch rise times arising from the cell model are within the physiological range of 40 to 110 ms (Garnett et al., 1979; Macefield et al., 1996) and show considerable impact on EMD, i.e., implying that muscles consisting mainly of fast-twitch fibers have lower EMD (cf. Figure 5E). Note that the motor unit / muscle twitch shapes are strongly dependent on the applied stretch (cf. Figure 3). This implies that experimentally observed twitch properties only reflect the contractile behavior of the muscle fibers if sufficient pre-stretch is applied to the muscle-tendon system (cf. section 3.1). In contrast to the twitch properties of the muscle fibers, the length dependence of the force-calcium relation is shown to have a minor impact on EMD. Similar results were obtained by Sasaki et al. (2011), who showed by employing mechanomyography that the duration of the chemo-electro-mechanical processes within the muscle fibers is independent from muscle length.

#### 4.2.4. Influence of Neural Control on EMD

By stimulating all muscle fibers synchronously the minimal theoretical EMD can be estimated, i.e., purely reflecting the properties of the muscle-tendon system, which, however, will most likely never be obtained during voluntary contractions. Comparing voluntary and evoked contractions, Hopkins et al. (2007) showed that EMD increases during voluntary contractions by more than a factor of two. To investigate the influence of neural control on EMD within this work, both the recruitment strength, i.e., how many muscle fibers are activated, and recruitment synchronicity are addressed. Thereby, especially the speed of recruitment, i.e., reflecting the time delay between the recruitment of two consecutive motor units, shows strong influence on EMD (cf. Figure 6B). Note that within this work each motor unit was stimulated once. For a realistic (progressive) recruitment scenario all motor neurons would continue to fire with frequencies of 5 to 40 Hz (cf. e.g., Kamen and Knight, 2004; Vecchio et al., 2019), however, this effect is believed to be negligible for the simulated conditions. Increasing the number of activated muscle fibers reduces EMD. However, the change in EMD caused by an increasing number of recruited muscle fibers decreases for higher recruitment strengths (cf. Figure 6A). Thus, EMD seems to be more sensitive to the speed of recruitment then to the applied recruitment strength. This is particularly interesting since neural control strategies like recruitment strength and synchronization can be modified by physical training, although the mechanisms are still under discussion (cf. e.g., Semmler and Nordstrom, 1998; Van Cutsem et al., 1998; Aagaard, 2003). While within this work all muscle fibers within a specific muscle are assumed to behave identical, the properties of muscle fibers such as e.g., the twitch shape, calcium dynamics and metabolism are believed to be closely related to the size of the corresponding motor unit (Duchateau and Enoka, 2011). Consequently, taking into account a continuous distribution of motor unit properties will most likely increase the influence of both recruitment strength and the speed of recruitment on EMD.

### 4.3. Outlook and Conclusion

Beside the properties that were related to EMD within this work, experimental studies suggested that also other factors such as the muscle and aponeurosis architecture or fatigue influence EMD (Nordez et al., 2009; Cè et al., 2013; Lacourpaille et al., 2013) and thus need to be addressed by further research. In principle, the multi-scale model can be adapted to capture fatigue, e.g., by employing a more detailed muscle fiber model (cf. e.g., Shorten et al., 2007), as well as to resolve both the geometry and the material behavior of aponeurosis. In order to quantify EMD for a specific joint, also the antagonistic muscles and the surrounding connective tissues have to be taken into account, which requires the application of detailed musculoskeletal system models (cf. e.g., Röhrle et al., 2017). Finally, while EMD is shown to be a property of a muscle-tendon system, it can be speculated that in order to specifically assess the functionality of internal muscle properties like excitation-contraction coupling alternative experimental methods, such as e.g., intramuscular pressure measurements (cf. e.g., Ateş et al., 2018), could be advantageous and need to be addressed by subsequent studies.

In summary, this study provides an *in silico* characterization of EMD, quantifying the contribution of multiple structures and functional properties to this phenomenon. Thereby, in relaxed conditions, i.e., when only little pre-stretch is applied to the muscle-tendon system, both the MTR as well as the material properties of muscle and tendon tissue significantly determine EMD. However, during physiological conditions, i.e., considering voluntary contractions, neural control is the most important factor influencing EMD. Thus, in order to reduce the risk of injuries caused by joint perturbations, improving the neural synchronicity seems to be the most promising prevention strategy.

## Data Availability Statement

The datasets generated for this study are available on request to the corresponding author.

## Author Contributions

TK, TS, and OR contributed to the conceptualization of the study. LS and TK derived the mathematical model, implemented the numerical model, and conducted the simulations. Further, all authors contributed to the analysis and the discussion of the results as well as the writing of the manuscript.

### Conflict of Interest

The authors declare that the research was conducted in the absence of any commercial or financial relationships that could be construed as a potential conflict of interest.
